# The impact of maternal nutrition on offspring’s risk of non-communicable diseases in adulthood: a systematic review

**DOI:** 10.7189/jogh.09.020405

**Published:** 2019-12

**Authors:** Jessie Pullar, Kremlin Wickramasinghe, Alessandro R Demaio, Nia Roberts, Karla-Maria Perez-Blanco, Katharine Noonan, Nick Townsend

**Affiliations:** 1 Centre on Population Approaches for NCD Prevention, Nuffield Department of Population Health, University of Oxford, Oxford, UK; 2 Evidence and Programme Guidance, Department of Nutrition for Health and Development, World Health Organisation, Geneva, Switzerland; 3 Health Library, Nuffield Department of Population Health, University of Oxford, Oxford, UK

## Abstract

**Background:**

A growing body of evidence suggests the impact of maternal nutrition plays a role in determining offspring’s risk of non-communicable diseases (NCDs), including heart disease (CVD), type 2 diabetes (T2DM), cancer and chronic obstructive pulmonary diseases (COPD). We conducted a systematic review to investigate this relationship.

**Methods:**

We systematically searched CINAHL, Cochrane Database of Systematic Reviews, Cochrane Register of Controlled Trials, Database of Abstracts of Reviews of Effects, MEDLINE, EMBASE, Web of Science Core Collection and Global Health for papers published before May 2016 (PROSPERO: CRD42016039244, CRD42016039247). Included studies examined the impact of maternal nutrition (diet, vitamin status and weight) on adult offspring’s NCD outcomes.

**Results:**

Of 23 501 identified citations, 20 met our inclusion criteria. Heterogeneity of papers required narrative synthesis. Included studies involved 1 939 786 participants. CVD: Four papers examined maternal exposure to famine during gestation, 3 identified a resulting increased risk of CVD in offspring. Five identified an increased risk of offspring CVD with increasing maternal weight. T2DM: Six studies investigated maternal exposure to famine during gestation; three identified an increase in offspring’s T2DM risk. Three found no increased risk; two of these were in circumstances where famine states persisted beyond pregnancy. Three papers found an increased risk of T2DM in offspring with increasing maternal BMI. CANCER: Four papers investigated maternal famine exposure during pregnancy – two identified a reduced risk of cancer in male offspring, and two an increased risk in female offspring. COPD: One study found low maternal vitamin D status was associated with reduced use of asthma medication.

**Conclusions:**

While there are indications that exposure to both famine (particularly when coupled with exposure to nutritional excess after birth) and maternal overweight during pregnancy is associated with offspring’s risk of CVD, T2DM and cancer, currently there is a lack of evidence to confirm this relationship. Despite the lack of conclusive evidence, these finding hold important research and policy implications for a lifecycle approach to the prevention of NCDs.

Malnutrition affects all regions of the world [1]. The double burden of malnutrition describes the co-existence of both under and over nutrition faced by individuals, households and whole populations [2]. The scale of this double burden is vast. In 2015, 462 million adults were underweight, 264 million women were affected by iron-amenable anaemia and 1.9 billion adults were overweight, with over 600 million of these obese [1]. Global action to address this double burden is essential in achieving the Sustainable Development goals and has gained political momentum through the 2014 Rome declaration on Nutrition and the UN Decade of Action on Nutrition from 2016-2025 [1,3].

Unhealthy diets are a leading risk factor for malnutrition, infant mortality and non-communicable diseases (NCDs) [3,4]. Rapid rates of globalisation and subsequent nutrition transitions has meant the burden of NCDs and over nutrition now supersedes the health burden of communicable disease and is accountable for 38 million deaths each year [3,5]. Cardiovascular disease (CVD), type 2 diabetes (T2DM), cancers and chronic obstructive respiratory diseases account for over 80% of this NCD burden [3]. Consequently these four diseases are the focus of the World Health Organisation's (WHO) Global Action Plan for the prevention and control of NCDs (NCD GAP) [3]. The NCD GAP also focuses prevention efforts towards addressing the four common behavioural risk factors for these NCDs – tobacco use, harmful use of alcohol, physical inactivity, and unhealthy diets in childhood and adulthood [3].

While maternal nutrition has long been recognised as a determinant on fetal growth, birth weight and infant mortality [6]. A growing body of evidence suggests this influence extends well beyond childhood. In fact, maternal nutrition may play a key role in their offspring’s risk of NCDs later in life. This is a profoundly important relationship, particularly in low and lower-middle income countries (LLMICs) who face the double burden of malnutrition, and 80% of the global NCD burden [7]. Due to their impact on ‘foetal programming’ both maternal under- and over-nutrition have been identified as a potentially critical component of the global NCD burden [6,8,9].

Currently WHO maternal nutrition targets and interventions focus on vitamin supplementation, breastfeeding and complementary feeding within the first 1000 days – from conception to two years of age [10]. Recently, the UN Secretary-General’s Global Strategy for Women’s and Children’s Health has also recommended integrating NCDs into maternal health programmes [11]. The NCD GAP supports this recommendation, calling for a life course approach to address NCDs [3].

To ensure targeted action and effectiveness within this life course approach a strengthened evidence base is required. This systematic review aims to add to that evidence base by examining the association between maternal nutrition (prior to conception, during gestation and during lactation) and offspring’s NCD outcomes. It is the first part of a two-part series. The accompanying review will examine the association between maternal nutrition prior to conception, during gestation and during lactation on the child’s risk of NCDs later in life.

## METHODS

Our review followed the Preferred Reporting Items for Systematic review and Meta-Analysis Protocols (PRISMA-P). The supporting PRISMA-P checklist is available in Appendix S1 in **Online Supplementary Document**. The current review presents a subset of the combined results from two PROSPERO registered protocols (CRD42016039244, CRD42016039247). The first outlines the methods for assessing maternal nutrition prior to and during pregnancy, while the second outlines the methods for assessing maternal nutrition during lactation. Deviation from proposed review outcomes was due to the size of magnitude of results which required division by outcome and age group. Ethics approval was not required for this review.

### Database and search strategies

We conducted a comprehensive literature search for citations published before 24^th^ May 2016. The search strategy was developed by a medical librarian in consultation with the review team. Search terms combined descriptors of pre-pregnancy, pregnancy, lactation and potential maternal nutrition factors with NCD morbidity and mortality descriptors in offspring, search terms are available in Appendix S2 in **Online Supplementary Document**. We conducted the search in English on the following nine databases: CINAHL, Cochrane Database of Systematic Reviews, Cochrane Register of Controlled Trials, Database of Abstracts of Reviews of Effects, MEDLINE, EMBASE, Web of Science Core Collection and Global Health. Additionally, we reviewed the reference lists of key reviews identified during screening.

### Inclusion criteria

Included research assessed the impact of maternal nutrition factors measured during, or prior to pregnancy and lactation on the offspring’s risk of CVD, T2DM, cancer and chronic obstructive pulmonary disease (COPD) at 18 years or older. Eligibility criteria are further outlined in **Table 1**.

**Table 1:** Eligibility criteria

Maternal nutrition measurement	Offspring NCD outcome
General population of mothers who:	The measurement of one of the following in offspring 18 years or older:
Had a nutrition indicator (including weight, weight gain, dietary intake, nutrition status) measured prior to/during pregnancy and lactation.	Cardiovascular Disease: Diagnosed coronary heart disease (heart attack), cerebrovascular disease (stroke), peripheral vascular disease, heart failure, congenital heart disease, cardiomyopathy.
Did not have any pre-existing chronic conditions (eg, HIV, diabetes, cardiovascular disease)	Diabetes Mellitus: Diagnosed type 2 diabetes.
Did not suffer preeclampsia, gestational diabetes or other adverse conditions during pregnancy.	Cancer: Diagnosis with any form of cancer
	Chronic respiratory diseases: Asthma, COPD, Pulmonary hypertension.
	Mortality outcome measures which are directly related to the above NCD’s such as heart attacks and stroke.

NCD – non-communicable disease, COPD – chronic obstructive pulmonary disease

No restrictions on publication language, location or study design were included. Studies were excluded if they did not measure a maternal nutrition factor, if they did not report on one of the predetermined outcome measures in offspring or if they were conducted on animals. Grey literature was excluded.

### Study selection and data extraction

Citations from the literature search were collated in Endnote and duplicates removed. Final results were exported into Excel for eligibility screening. Using the eligibility criteria in **Table 1** – JP, KP, KN and AF independently screened titles and abstracts. The Cohen's kappa statistic was calculated at 10% intervals (approximately every 2000 papers) to ensure consistency in screening. Once the Cohen's kappa statistic exceeded 0.75 indicating “excellent agreement”, KP, KN and AF screened all remaining records. Uncertainties were brought to JP, KW and NT and disagreements resolved by group consensus. JP then reviewed all citations selected for full text review, discussing any uncertainties with KW and NT.

Following full text review, the selected studies reference lists were examined for any relevant studies. Data extraction was then conducted independently by JP on all included. Data collection involved the extraction of maternal nutrition measures, country the study was conducted in, participant characteristics, offspring NCD related outcomes (risk of NCDs), offspring age, limitations, study design, details on adjustment for confounders and quality assessment measures.

### Quality assessment

The Newcastle-Ottawa scale (NOS) was used by JP and KW for assessing the risk of bias in cohort studies, with validated, modified versions used for cross-sectional and case-controlled studies (Appendix S3 in **Online Supplementary Document**) [12].

### Synthesis of results

Due to the heterogeneity of the design and outcome measures in the included studies, a meta-analysis was not possible. Narrative synthesis of data was conducted by JP.

## RESULTS

**Figure 1:**
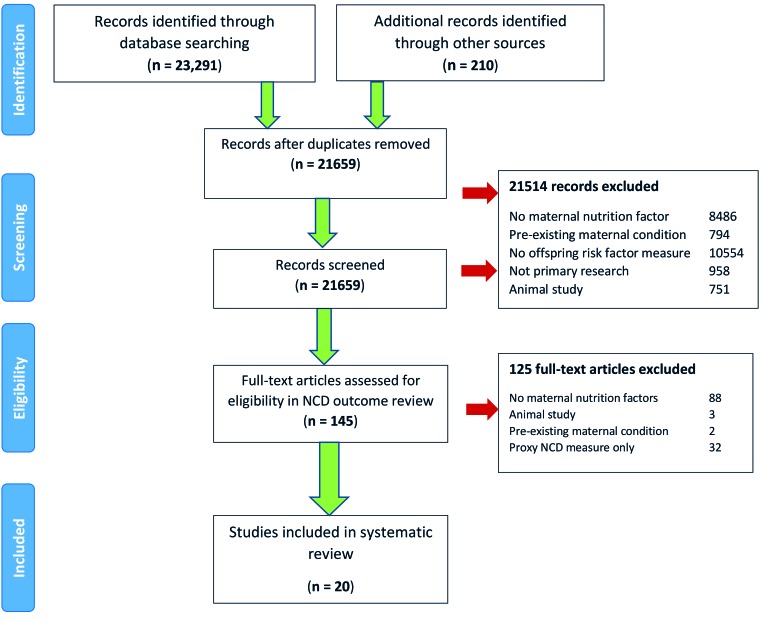
PRISMA flow diagram.

Our initial search returned 23 501 records. After excluding 23 356 records based on title and abstract screening, 145 papers were included for full-text review. Twenty studies involving 1 921 836 participants were included (**Figure 1**).

**Figure 2:**
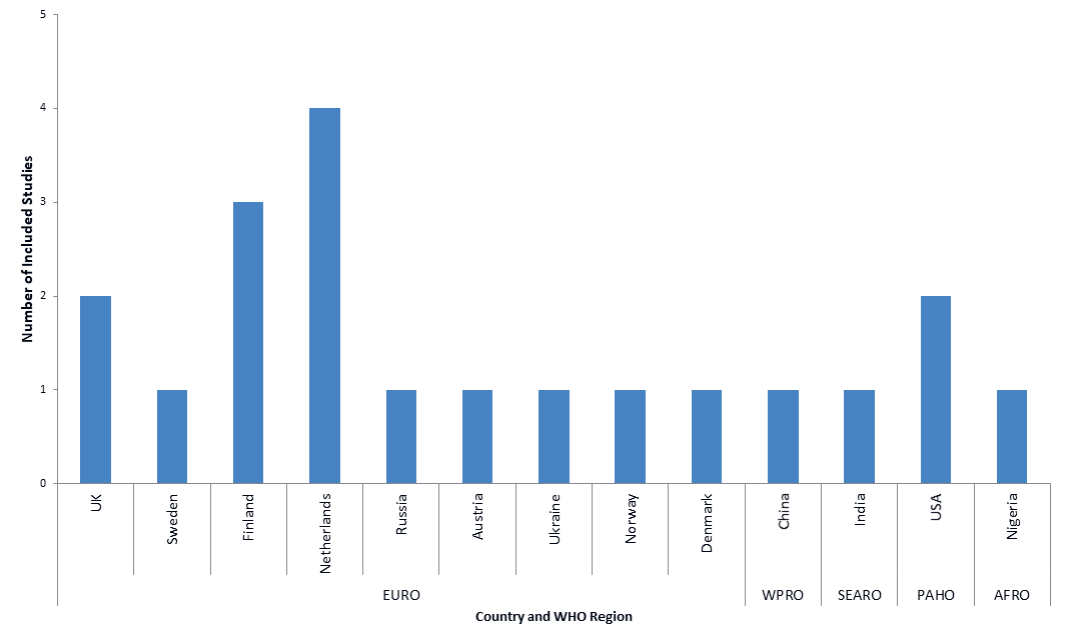
Countries represented by included studies according to WHO region.

Twelve studies were retrospective cohorts, four case-control and three cross-sectional and. Nine studies (41%) were published before 2010. **Figure 2** shows the country of origin of included studies according to WHO region. The majority (15 studies, 75%) came from the European region, with 20% from the Netherlands. No studies from the WHO’s Eastern Mediterranean region met inclusion criteria. Of the 13 countries represented, none were low income countries, three were lower-middle income countries, two were upper-middle income and the remaining eight (62%) were high income countries.

Included studies involved nine different cohorts, four studies (20%) involved offspring of the Dutch Famine Birth Cohort (DFBC), three examined the Helsinki Birth Cohort (HBC), and two examined the Aberdeen Birth Cohort (ABC). Nine studies reported on the impact of maternal nutrition on CVD, nine on T2DM, six on cancer and one on COPD. The age of offspring at the time of follow up ranged from 10-69 years (with one study including participants younger than the inclusion cut off due to an inability to separate results from older subjects). Of the included studies, 10 (50%) examined the impact of maternal exposure to famine, seven (36%) the impact of maternal body weight, two gestational weight gain and one maternal vitamin D status, anaemia and coffee intakes impact on offspring health outcomes. No studies evaluated the impact of maternal macronutrient intake. All studies measured these influences during gestation, with one also measuring the impact of pre-pregnancy weight. No included studies measured the impact of maternal nutrition during lactation.

**Figure 3:**
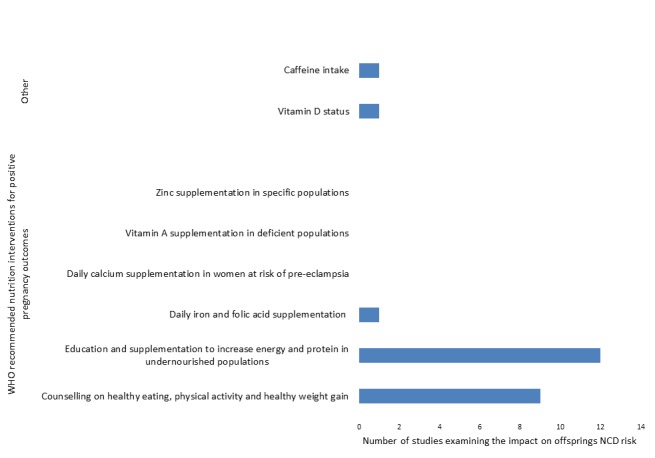
Number of included studies reporting outcomes related to WHO recommendations for nutrition interventions in antenatal care.

When compared to current WHO recommendations for nutrition interventions during antenatal care [13], findings from included studies are consistent with existing recommendations, namely counselling on healthy diets and healthy weight/ weight gain and the prevention of undernutrition through counselling and the provision of protein and energy supplementation (**Figure 3**).

### Cardiovascular disease

**Table 2** shows nine cohort studies examined CVD outcomes in 118 185 offspring. All were conducted in the European region and the majority (56%) assessed offspring who were aged 50 years or older. Five examined the impact of maternal pre-pregnancy or pregnancy weight, four the impact of exposure to famine during pregnancy and one examined the impact of gestational weight gain. There did not appear to be an increased likelihood of identifying a significant increase in CVD outcomes due to maternal nutrition exposure based on the age of offspring when measured.

**Table 2:** Impact of maternal nutrition on offspring’s CVD related outcomes

Authors (Year), country, study type	Subjects (n, age range)	Data collection method	Maternal nutrition exposure	Outcome
Reynolds RM, et al [14] (2013). United Kingdom, Retrospective Cohort	37 709 (7134 exposed to ow/ob), 34-61 y	Maternal BMI taken at first ANC within the ABC. Linked to national death and morbidity records	Maternal weight during pregnancy (overweight 25-29.9, obesity >30)	Hazard ratio for CVD event related hospital admissions in offspring: Maternal BMI <18.5 kg/m^2^ = 0.90 (95%CI = 0.63-1.00); Maternal BMI 25-29.9 kg/m^2^ = 1.15 (95% CI = 1.04-1.26); Maternal BMI>30 kg/m^2^ = 1.29 (95% CI = 1.06- 1.57)
				Hazard ratio for cerebrovascular disease in offspring: Maternal BMI <18.5 kg/m^2^ = 0.64 (95% CI = 0.20-2.05); Maternal BMI 25-29.9 kg/m^2^ = 1.61 (95% CI = 1.10-2.36); Maternal BMI>30 kg/m^2^ = 1.54 (95% CI = 0.69-3.42)
				Maternal overweight and obesity were associated with a significant increase in CVD related hospital admissions in offspring. Maternal overweight was also associated with a significant increase in cerebrovascular disease in offspring. No significant association was found for angina, myocardial infarction, stroke or peripheral artery disease in offspring. Risk of premature death (<55 y) was 40% higher for offspring of obese mothers (HR = 1.4, 95% CI = 1.17-1.68).
Bhattacharya S, et al [15] (2015). United Kingdom, Retrospective Cohort	N = 3781 43-49 y	The ABC of the 1950s – linked with local obstetric and national vital statistics and hospital clinical data sets	Gestational weight gain (GWG)	For offspring whose mothers had a rate of GWG of ≥1 kg/week demonstrated a significantly higher risk of suffering a cerebrovascular event (aHR = 2.70, 95% CI = 1.19-6.12). The risk of suffering a cardiovascular event (aHR = 1.37, 95% CI = 0.59-3.18), or mortality (aHR = 0.47, 95% CI = 0.07, 3.10) was not significantly higher. There were no significant associations between lower rates of GWG and CVD outcomes.
Eriksson JG, et al [16] (2011). Finland, Retrospective Cohort	N = 6975 men (655 cases of CHD), 58-63 y	HBC – linked to offspring hospital admissions and deaths using national identification numbers	Maternal weight during pregnancy	For mothers with height >160 cm and BMI>25 kg/m^2^ during pregnancy: Offspring who had a low ponderal index ≤25 kg/m^3^ had an elevated risk of CHD (HR = 1.9 (95% CI = 1.1-3.2), *P* for trend with reducing ponderal index trend = 0.01; Offspring who had a low placental weight ≤550 g had an elevated risk of CHD (HR = 2.2, 95% CI = 1.3-4.0), *P* for trend with reducing placental weight = 0.002
				Of mothers with height >160 cm and BMI <25 kg/m^2^ during pregnancy: Offspring with placental/ birth weight (%)>20.5% had an increased CHD risk (HR = 1.8, 95% CI = 1.1-3.0), *P* for trend with increasing percentage = 0.01
				These relationships were not apparent according to BMI in women under <160 cm
Forsen T, et al [17] (1997). Finland, Retrospective Cohort	3302 men, 47-71 y	Data from men within the HBC. Linked to national mortality register	Maternal BMI in late pregnancy	Risk of CHD rose with every standard deviation increase in mothers BMI (HR = 1.24, 95% CI = 1.10-1.39, *P* = 0.0004), mean BMI was 26.9
				For mothers with height <1.58 m the risk of CHD in offspring rose with increasing BMI (BMI>30: mortality ratio 171 vs BMI <25: mortality ratio 56, *P* = 0.006)
				No significant association in mothers >1.58 m tall.
Eriksson JG, et al [18] (2014). Finland, Retrospective Cohort	N = 13 345, 70-80 y	Offspring of the HBC – maternal BMI measured prior to delivery and linked to offspring’s national health records	Maternal overweight in pregnancy	Offspring’s risk of CVD increased significantly with increasing maternal BMI (BMI ≤24 kg/m^2^ HR = 1.0 vs BMI ≥28 kg/m^2^ HR = 1.13) with HR = 1.026 trend per kg/m^2^, 95% CI = 1.010-1.042, *P* for trend = 0.002)
				Offspring’s risk of CHD increased significantly with increasing maternal BMI (BMI ≤24 kg/m^2^ HR = 1.0 vs BMI ≥28 kg/m^2^ HR = 1.13) with HR = 1.030 trend per kg/m^2^, 95% CI = 1.010- 1.050, *P* for trend = 0.003)
				Offspring’s risk of CHD increased significantly with increasing maternal BMI (BMI ≤24 kg/m^2^ HR = 1.0 vs BMI ≥28 kg/m^2^ HR = 1.13) with HR = 1.030 trend per kg/m^2^ = , 95% CI = 1.010- 1.050, *P* for trend = 0.003)
Painter RC, et al [19] (2006). Netherlands, Case Control	N = 2414 (385 exposed), 50-58 y	Medical records of mothers within DFBC. Offspring identified by council records and results measured during hospital visit	Maternal exposure to famine during early, mid, late pregnancy (<1000 cal/d)	The only significant increase in CAD was seen in offspring whose mothers were exposed in early gestation (cumulative incidence 13% vs 8% in non-exposed, aHR = 1.9 (95% CI = 1.0-3.8, *P* < 0.05) and the age of onset was two years younger (47 y vs 49 y, *P* < 0.05)
				Compared to mothers not exposed to famine, maternal weight gain in the last trimester, and weight at the end of pregnancy was significantly lower (by 1 kg and 2 kg respectively, *P* < 0.005).
Bygren LO, et al [20] (2000). Sweden, Retrospective Cohort	N = 7572 (exposed), 40-70 y	Subjects born in the parish of Skellefteå, Sweden between 1805 and 1849 and living there at the age of 40.	Exposure to famine during pregnancy	The relative risk (RR) of sudden death was almost doubled for those whose mothers were exposed to famine in early gestation, and a good harvest in late gestation (RR = 1.99, 95% CI = 1.22-3.25)
				Similar risk was evident where there was plentiful food supply in early gestation and famine exposure in late gestation (RR = 1.68, 95% CI = 1.03-2.75)
				Poor food availability throughout pregnancy was not associated with CVD related sudden death (RR = 0.98, 95% CI = 0.55-1.58).
Van Abeelen AF, et al [21] (2012). Netherlands, Retrospective Cohort	1991, 18-64 y	Offspring from the DFBC (Linked to Statistics Netherlands mortality data).	Exposure to famine during pregnancy	206 subjects (10%) had died at the time of analysis
				Women exposed to famine in early gestation had a significantly higher risk of cardiovascular mortality (HR = 4.6, 95% CI = 1.2-17.7, *P* = 0.03)
				Men exposed to famine in early gestation showed a non-significant reduction in cardiovascular mortality (HR = 0.9; 95% CI = 0.3, 3.1, *P* = 0.90)
				Exposure to famine in mid and late gestation was not associated with significantly increased morality from any cause in men and women.
Ekamper P, et al [22] (2015). Netherlands, Retrospective Cohort	41 096 men (22 952 exposed), 63 y	Male conscripts born between into the DFBC. Linked to military health records.	Exposure to famine during pregnancy (<900 kcal/d)	CVD accounted for 28.3% of recorded deaths but this did not indicate a significant increase in CVD related mortality following gestational exposure to famine (HR = 1.07, 95% CI = 0.91-1.26), or pre-pregnancy exposure (HR = 1.13, 95% CI = 0.94-1.36)
				There was no significant increase in cerebrovascular disease following maternal exposure to famine before or during gestation (HR = 1.02, 95% CI = 0.72-1.44).

aHR – adjusted hazard ratio, ANC – ante-natal clinic, ABC – Aberdeen Birth Cohort, BMI – body mass index, CAD – coronary artery disease, Cal – calorie, CHD – coronary heart disease, CI – confidence interval, CVD – cardiovascular disease, DFBC – Dutch Famine Birth Cohort, GWG – gestational weight gain, HBC – Hamamatsu Birth Cohort, HR – heart rate, y – years

A large analysis of the ABC (n = 7134) found that at 50 years of age offspring of overweight and obese mothers during pregnancy had an increased risk of CVD events requiring hospital admission (15% and 29% respectively) [14]. The largest increase was seen for cerebrovascular disease (61% and 54% increased risk respectively). This is consistent with an earlier examination of the same cohort which found a 19% increase in cerebrovascular disease risk for offspring whose mothers gained more than 1 kg/week [15]. No other significant associations were identified. Within this analysis it was also identified that offspring’s lifestyle factors such as adult smoking, social class, BMI and diabetes status demonstrated a larger statistically significant impact on CVD outcomes [15].

An analysis specific to men born in the HBC found no association between mothers’ weight and offspring’s risk of coronary heart disease (CHD) [16]. When mothers were divided based on height (over or below 160 cm), a significant association was found for increased risk of CHD in men of taller mothers. This association was mediated by mens low ponderal index (<25 kg/m^3^ hazard ratio (HR) = 1.7, 95% confidence interval (CI) = 1.2-2.5), with p for trend in increased CHD risk with lower ponderal index *P* = 0.007). Ponderal index is a measure of thinness- similar to BMI measures but used in children under 2 years of age, it is calculated by dividing body weight by height cubed.

When further analysed based on mothers BMI, no significant association was found for shorter mothers, though in taller mothers, offspring’s CHD rose in mothers with a BMI over 26 kg/m^2^ (HR = 1.25, 95% CI = 1.10-1.42) per 40 cm^2^ decrease in placental surface area (*P* for trend = 0.0007) [16]. For taller mothers, whose BMI was over 26 kg/m^2^ offspring who had an increased risk of CHD with increasing thinness (measured by centimeter increase in the difference between length and breadth of placental surface). Low subject numbers within subgroups mean this analysis had low power. An earlier examination of the same male cohort found no significant increase in CHD related mortality for offspring of taller mothers [17]. This analysis identified an increased risk of CHD mortality for offspring of mothers who were shorter than 158 cm and had a BMI>30 in comparison to mothers with a healthy weight (mortality ratio 171 vs 56, *P* = 0.006) [17]. The final paper identified a significant, 13% increase in CVD and CHD risk for the female offspring of mothers whose BMI was over 28 kg/m^2^ [18]. A small increase in risk was also seen for stroke in offspring of mothers whose BMI was over 28 kg/m^2^. There was a significant trend for increased risk of CVD, CHD and stroke in offspring with each unit increase in maternal kg/m^2^, *P* < 0.05 [18].

Of the four studies investigating the impact of maternal exposure to famine during pregnancy, three found an increased risk of CVD in offspring [19-21]. One found maternal exposure to the Dutch famine was associated with a 5% increased prevalence of coronary artery disease (CAD) in offspring over 50 years old, and average age of onset two years younger in those exposed in early gestation compared to non-exposed [19]. An ancient Swedish cohort also identified an increased risk of sudden death from cerebrovascular and cardiovascular disease in offspring of mothers exposed to food shortages in early or late gestation- though no significant association for those exposed to food shortages throughout pregnancy [20]. Female subjects exposed to the Dutch famine during early gestation demonstrated a hazard ratio of CVD mortality 4.6 times greater than unexposed subjects, though no significant association was found for men, or those exposed in mid to late gestation [21]. Analysis of a large, DFBC sample of male subjects found no significant association with CVD mortality [22].

### Type 2 diabetes mellitus

**Table 3** shows nine papers examined the impact of maternal nutrition on T2DM in offspring. Six examined the impact of exposure to famine during gestation, and three investigated the impact of maternal obesity. The majority (56%), were from the European region and measured outcomes in offspring who were 50 years of age or older (56%). There did not appear to be a consistent association between the age of offspring’s and the identification of a significant increase in T2DM due to maternal obesity or exposure to famine.

**Table 3:** The impact of maternal nutrition factors on type 2 diabetes mellitus (T2DM) prevalence in offspring

Reference (year). country, study type	Subjects (n, age range)	Data collection method	maternal nutrition factor	Outcomes
Li, Y et al [23] (2010). China, Retrospective Cohort	7874 (1005 exposed to famine), 45-69 y	Adults born between 1954-1964 in rural communities exposed to the Chinese famine. Follow-up data available from China's 2002 National Nutrition and Health Survey	Exposure to the Chinese famine during pregnancy	T2DM prevalence in exposed vs non-exposed (2.01% vs 1.37%). Risk of T2DM not significantly higher in exposed cohort (aOR = 1.43, 95% CI = 0.53-3.87).
Stanner et al [24] (1997). Russia, Cross-Sectional Study	549 (169 exposed to famine), 52 y	Offspring identified through the register for the Society of Children of the Siege and invited to attend to attend endocrinology clinic for measurement	Exposure to the Leningrad siege during pregnancy (<300 carbohydrate calories/d)	No significant difference in the prevalence of known T2DM with intrauterine exposure (mean 2.3% vs 3.6%) newly diagnosed T2DM (1.8% vs 2.7%), impaired glucose tolerance (9.6% vs 8.6%) compared to unexposed. No significant differences between prevalence rates in those exposed during gestation or infancy.
Hult M, et al. [25] (2010). Nigeria, Retrospective Cohort	1339, 39-41 y	Cohort of Igbo adults exposed to Biafran famine in gestation and early infancy. Convenience sample of offspring taken from six major market places.	Exposure to the Biafran famine during pregnancy and early infancy	Fetal-infant exposure to famine was associated with a significant increase in diabetes (OR = 3.11, 95% CI = 1.14-8.51), though when adjusted for BMI this was no longer significant (OR = 2.56, 95% CI = 0.92-7.17).
Lumey, LH et al [26] (2015). Ukraine, Retrospective Cohort	1 464 174 (599 759 exposed), 63-71 y	Individuals born between 1930 and 1938 from the 2001 Ukraine national census as the reference population. Ukraine national diabetes register 2000-08 for T2DM diagnosed at aged >40 y.	Exposure to the Ukraine famine during pregnancy	Higher risk of T2DM in subjects born in regions with severe famine (aOR = 1.23, 95% CI = 1.07-1.40), and extreme famine (aOR = 1.51, 95% CI = 1.35-1.69) (combined for all regions and birth years) compared to individuals not exposed to famine (OR = 1.00, 95% CI = 0.91-1.09).
Thurner S, et al [27] (2013). Austria, Cross-Sectional Study	325 000, 62-91 y	Database of the Main Association of Austrian Social Security Institutions – linked birth year with health care outpatient as well as inpatient care in Austria years of famine 1918-1919, 1938, 1946-1947.	Exposure to famine during pregnancy	Risk of developing T2DM was ∼ 13% higher in males and 16% in females than the national average for those born in the 1919-1921 famine compared to those born outside of famine. Excess risk of diabetes was 9% males/ 8% females for offspring born in 1938 famine, and 5% males, 3% female for those of 1946–1947.
Ekamper P, et al [22] (2015). Netherlands, Retrospective Cohort	41 096 men (22 952 exposed), 63 y	Male conscripts included in the DFBC. Linked to military records of health and mortality.	Exposure to famine during pregnancy (<900 kcal/d)	Of 5011 deaths T2DM accounted for 115 (2.3%) deaths. There was no increased risk of T2DM related mortality following maternal exposure to famine prior to or during pregnancy (HR = 1.61, 95% CI = 0.91-2.86, *P* > 0.05).
Fall CH, et al [28] (1998). India, Cross-Sectional Study	506 (76 with T2DM), 45-63 y	Detailed obstetric records from Mysore hospital for pregnancies between 1934-1953. Cohort traced and recruited for hospital check up.	Mother's BMI during pregnancy	76 offspring (15%) diagnosed with T2DM.
				Offspring incidence of T2DM increased with increasing maternal weight (10% with mothers under 43kg, 24% T2DM prevalence with mothers with maternal weight >49kg)
				Significant trend for diagnosis with T2DMs in offspring whose mothers had a higher body weight during pregnancy (*P* = 0.004). T2DM was also related to ponderal index.
Dabelea D, et al [29] (2008). USA	158, 10-22 y	Offspring with T2DM recruited from the Diabetes in Youth Study. Maternal obesity recorded by mothers self-reported recall.	Maternal obesity during pregnancy (BMI ≥25 kg/m^2^)	79 subjects diagnosed with T2DM. Subjects with T2DM were more likely to have been exposed to maternal obesity during pregnancy (57% vs 27.4%, *P* < 0.0001), exposure to maternal obesity was independently associated with T2DM (aOR = 2.8, 95% CI = 1.5-5.2, *P* < 0.0001).
Eriksson JG, et al [18] (2014). Finland, Retrospective Cohort	N = 13 345, 70-80 y	Offspring of the HBC – maternal BMI measured prior to delivery and linked to offspring’s national health records.	Maternal overweight in pregnancy	Offspring’s risk of T2DM was significantly associated with increasing maternal BMI above 24 kg/m^2^ (BMI ≤24kg/m2 HR = 1.0 vs BMI ≥28 kg/m^2^ HR = 1.20, *P* < 0.05) with a HRfor trend per kg/m^2^ = 1.040 (95% CI = 1.013-1.068, P for trend = 0.004).

aOR – adjusted odds ratio, HR – hazard ratio, BMI – body mass index, Cal – calorie, CVD – cardiovascular disease, HBC – Hamamatsu Birth Cohort, HR – heart rate, y – year

Examination of exposure to the Chinese famine during gestation on offspring’s T2DM risk found no significant difference in the prevalence of T2DM between exposed and non-exposed cohorts, regardless of severity of famine exposure [23]. A small cohort of 549 subjects, examining famine exposure during pregnancy as a result of the 1941-1944 Leningrad siege also failed to find a significant impact on offspring’s T2DM risk [24]. In contrast, a Nigerian cross-sectional analysis found the odds ratio of T2DM was over three times greater in offspring exposed to the Biafran famine during gestation and early infancy [25]. Though this relationship was not significant once adjusted for offspring’s BMI, exposure to the famine was itself associated with increased BMI in offspring [25]. In a large cohort, adults exposed to the Ukraine famine of 1932-33 identified a dose-response relationship between severity of exposure during gestation and T2DM risk [26]. Those exposed to extreme famine during gestation showed a 47% increase in T2DM risk (adjusted odds ratio (aOR) = 1.47, 95% CI = 1.37-1.58), and a 26% increased risk in those exposed to severe famine (aOR = 1.26, 95% CI = 1.14-1.39) in comparison to non-exposed cohorts [26]. A population analysis of all diagnosed cases of T2DM in Austria found an increased risk of T2DM in offspring born immediately after periods of famine- compared to those born outside of these periods, the significance of these relationships was not reported [27]. Analysis of a small, male DFBC subset found no significant increase in diabetes related mortality following maternal famine exposure [22].

Three studies reported on the impact of maternal obesity during pregnancy and T2DM. One small Indian cohort found a significant trend for increasing T2DM risk in offspring with increasing maternal weight during pregnancy [28]. The second American study found subjects with T2DM were significantly more likely to have mothers who were overweight during pregnancy [29]. Comparing the prevalence of maternal obesity against controls. The largest study examining this association involving the HBC found offspring’s risk of T2DM was significantly associated with increasing maternal BMI with a 4% increase in T2DM risk for every kg/m^2^ unit increase in maternal weight (*P* for trend = 0.004) [18].

### Cancer

**Table 4** shows six studies examined the impact of maternal nutrition on offspring’s cancer outcomes. The majority (83%) were conducted on European cohorts, 50% involved offspring who were aged 50 years or older. Four studies examined the impact of maternal exposure to famine. Two studies examined this relationship in male subjects, one found males showed a reduced prevalence of testicular cancer following lower maternal weight during pregnancy due to national food shortages [30]. The other, from the DFBC, found no significant increase in cancer related mortality as a result of maternal exposure to famine -but did identify a 15% reduction in the risk of malignant neoplasms [22]. Analysis of the DFBC cohort identified a 2-fold increase in female offspring’s cancer related mortality associated with maternal exposure to famine in early gestation (HR = 2.3, 95% CI = 1.1-4.7, *P* = 0.03), as well as an 8-fold increase in breast cancer mortality. No significant association was found for male offspring, or those exposed during mid or late gestation [21]. Examination of exposure to famine during gestation on offspring’s breast cancer risk found an increased risk (HR = 2.6, 95% CI = 0.9-7.7) [19].

**Table 4:** The impact of maternal nutrition on offspring cancer related outcomes

Reference (year). country, study type	Subjects (n, age range)	Data collection method	Maternal nutrition exposure	Outcome in offspring
Aschim EL, et al [30] (2005). Norway, Case Control Study	1790, 21-45 y	Male offspring from women giving birth at the National Hospital in Oslo, Norway each month from 1931-1955. Linked to national testicular cancer cases register.	Maternal exposure to famine (impact on BMI)	Testicular Cancer (TC): Reduction in maternal weight of between 2-3 kg from 1941-1945 compared to 1951-1955 (BMI 26.3 vs 27.9) correlated with a reduction in TC incidence (465 vs 535, Spearman’s rho = 1.00, *P* < 0.01; Pearson r = 0.95, *P* = 0.02).
Ekamper P, et al [22] (2015). Netherlands, Retrospective Cohort	41 096 men (22 952 exposed), 63 y	Male conscripts born between 1944 and 1947 in famine affected areas by the DFBC. Linked to military records of health and mortality.	Exposure to famine during pregnancy (<900 kcal/d)	Cancer Related Mortality and Malignant Neoplasms: 5011 subjects had died at the time of analysis. Cancer accounted for 38.7% of recorded deaths. There was no significant increase in cancer related mortality following gestational exposure to famine (HR = 0.98, 95% CI = 0.87-1.11). Reduced risk of malignant neoplasms in offspring following maternal exposure prior to pregnancy was found (HR = 0.85, 95% CI = 0.74- 0.99). Highest number of neoplasms recorded for digestive organs (30%) and respiratory organs (29%). Famine exposure in the first trimester of gestation increases mortality from other natural causes (HR = 1.24, 95% CI = 1.03-1.49) – includes diabetes and cerebrovascular disease.
Van Abeelen AF, et al [21] (2012). Netherlands, Retrospective Cohort	1991, 18-64 y	Offspring from the DFBC. Linked to Statistics Netherlands mortality data.	Exposure to famine during pregnancy	Cancer Related Mortality: 206 (10%) had died at the time of analysis. Women exposed to famine in early gestation had a significantly higher risk of cancer mortality (HR = 2.3, 95% CI = 1.1-4.7, *P* = 0.03), and breast cancer mortality (HR = 8.3, 95% CI = 1.1-63.0, *P* = 0.04). Men exposed to famine in early gestation showed a non-significant reduction in cancer mortality (H = 0.3, 95% CI = 0.0-1.9, *P* = 0.19). Exposure to famine in mid and late gestation was not associated with significantly increased cancer related morality in men or women.
Painter RC, et al [19] (2006). Netherlands, Case Control Study	475 women, 61-62 y	Offspring from the DFBC (Linked to Statistics Netherlands mortality data.).	Exposure to famine during pregnancy	Breast Cancer: Overall, when adjusted for maternal body mass index, the hazard ratio of breast cancer in offspring of women exposed to famine during pregnancy was significant higher (HR = 4.0, 95% CI = 1.1-14.5), particularly for those exposed in early gestation (HR = 7.1, 95% CI = 1.6-32.0).
Sanderson M, et al [31] (1998). USA, Case Control Study	946 (510 cases), 45 y	Data collected from the mothers of women in two population-based case–control studies of breast cancer in women under the age of 45 y	Gestational weight gain	Breast Cancer: Gestational weight gain of 11-15 kg was associated with an increased risk of breast cancer (OR = 1.5, 95% CI = 1.1- 2.0); however, women whose mothers gained 15.5 kg or more were not at an increased risk. No association between maternal coffee consumption or anaemia status was found.
Eriksson JG, et al [18] (2014). Finland, retrospective cohort	N = 13 345, 70-80 y	Offspring of the HBC– maternal BMI measured prior to delivery and linked to offspring’s national health records	Maternal overweight in pregnancy	Cancer Incidence and cancer related death: Offspring’s risk of cancer incidence was not significantly associated with maternal BMI (HR for trend per kg/m^2^ = 1.017, 95% CI = 0.998-1.036, *P* = 0.08). There was also no significant association found for maternal BMI and offspring risk of cancer related death (HR for trend = 1.013, 95% CI = 0.983-1.044, *P* = 0.4).

HR – hazard ratio, CI – confidence interval, BMI – body mass index, y – year

A small cross-sectional analysis identified an increased risk of breast cancer in the offspring of women who gained 11-15 kgs during gestation (OR = 1.5, 95% CI = 1.1-2.0) [31], however there was no significant increase in risk for offspring whose mothers gained more than 15 kg. Maternal anaemia status and coffee intake failed to show a significant relationship with offspring’s breast cancer risk. Within the HBC no significant association between maternal BMI and cancer incidence, or related mortality, was found [18].

### Chronic obstructive pulmonary disease

**Table 5** shows only one relatively small cohort from Denmark investigated the impact of maternal nutrition on chronic obstructive respiratory disorders, specifically examining maternal vitamin D levels on asthma in offspring (126 cases of asthma). A significant reduction in asthma medication use was seen in the offspring of mothers with the lowest quartile of maternal 25(OH)D concentration, though no significant association with higher vitamin D levels, or vitamin D levels and asthma related hospitalizations [32].

**Table 5:** Impact of maternal nutrition on the incidence of chronic obstructive pulmonary disease in offspring

Reference (year). country, study type	Subjects (n, age range)	Data collection method	Maternal nutrition exposure	Outcome, HR (95% CI)
Hansen S, et al [32] (2015). Denmark, Retrospective Cohort	840 (126 asthmatics), 20-25 y	Mothers recruited between 1988 and 1989 for The Danish Fetal Origins Cohort during third trimester of pregnancy. The register of Medicinal Product Statistics Data was used to establish asthma prescriptions.	Maternal vitamin D status (25(OH)D concentration) during pregnancy	Significant reduction in useof asthma medication in offspring of mothers with lowest vitamin D levels (HR = 0.57, 95% CI = 0.35-0.95) compared to reference group. No significant association was found in offspring of mothers with the highest vitamin D levels (HR = 1.02, 95% CI = 0.61-1.17)
				No significant trend for asthma mediation use according to maternal vitamin D levels (*P* = 5.09)
				High maternal vitamin D levels (≥125 nmol/L) was not associated with asthma hospitalisations in offspring (HR = 1.81, 95% CI = 0.78-4.16).

HR – hazard ratio, CI – confidence interval, y – year

## DISCUSSION

The current review demonstrated the low number of studies investigating the association between maternal nutrition and offspring’s NCD risk, though available papers suggest both maternal obesity and undernourishment play an important role in offspring’s risk of CVD and T2DM in adulthood. There is also some evidence that maternal undernourishment during pregnancy may increase female offspring’s risk of breast cancer and cancer related mortality, while actually reducing male offspring’s risk of cancer. These results are based primarily on European cohort studies.

### Cardiovascular disease

All five studies investigating the impact of higher maternal BMI on CVD outcomes in offspring identified an increased risk. The largest cohorts investigating this relationship found a significant increase in CVD events between 13%-29% [14,18] amongst offspring of mothers with recorded BMI values over 25 kg/m^2^. Within the HBC there was also a significant trend identified for increasing risk of CVD, CHD and to a lesser extent stroke for each standard deviation point increase in BMI above 24 kg/m^2^ [18]. Similar trends were found for CHD risk in offspring by Forsen et al [17], while an increased risk of CHD was found in male offspring of taller mothers who had a BMI over 25 kg/m^2^, a relationship that was reliant of offspring who were also thin at birth (measured by low ponderal index) [16]. An increased risk of cerebrovascular diseases was identified in offspring of mothers who had a BMI value over 25 kg/m^2^ [14] and mothers who gained over 1 kg a week during gestation [15]. Though all studies identified significant increases in risks, none indicated an increase in mortality and impacts were generally specific to cardiovascular events, coronary heart diseases and cerebrovascular disease. The largest analysis by Reynolds et al. found no relationship with the risk of angina, myocardial infarction, stroke or peripheral artery disease in offspring [14].

Maternal obesity and excessive gestational weight gain has previously been associated with a host of adverse birth outcomes, including macrosomia and childhood obesity [33,34]. A recent systematic review and meta-analysis found that once publication bias was accounted for, no protective effect was apparent for maternal obesity against low birth weight. Instead, the risk of having a very (<1500 g RR = 1.61, 95% CI = 1.42-1.82), or extremely (<1000 g RR = 1.31, 95% CI = 1.08-1.59) low birth weight infant was actually increased in overweight and obese mothers [35]. This is consistent with findings of the impact of maternal weight on CVD outcomes in offspring being linked to thinness at birth which is an indicator of intrauterine growth restriction [16]. Two cohorts from the United Kingdom also identified higher rates of CHD [36] and death from ischemic heart disease [37] amongst men who had a low birth weight.

Within the four included studies, three found exposure to famine during gestation increased the risk of CVD outcomes. Each of these examined the timing of exposure to famine and found exposure during early gestation was associated with an increased risk of CAD [19], sudden death from CVD [20] and a 4-fold increase in CVD for women [21]. Within the Swedish cohort it was found that radical changes to nutrient availability during gestation, rather than chronic famine exposure throughout gestation, increased offspring’s risk [20]. The largest cohort also failed to identify an increased risk of CVD related mortality as a result of famine exposure during gestation [22].

### Type 2 diabetes mellitus

The six papers examining the impact of maternal exposure to famine during pregnancy on offspring’s risk of T2DM found mixed results. While cohorts from Nigeria [38], Austria [27] and the Ukraine [26] found significant increases in offspring’s risk, ranging from 3% to a 3-fold increase – other large cohorts from Russia [24], China [23] and the Netherlands [22] found no increased risk. In studies which did not find an association, contextual factors may explain this null finding. For example, the DFBC subset analysis suffered from small sample sizes and limited power [22]. Within analysis of subjects exposed to the Chinese famine the timing of exposure was not well defined [23], this was also the case within the Russian cohort as it is thought that exposure to famine was not confined to pregnancy due to ongoing food shortages with Leningrad once the siege had lifted [24]. These are important considerations when results are compared to the positive findings of the larger studies from the Ukrainian and Austria which suggest a dose-response relationship with famine exposure in gestation and T2DM, particularly for those exposed in early gestation. Examination of the Ukraine famine found a dose response relationship with exposure to T2DM leading to an increased risk ranging between 26%-47% [26]. However, the diabetes cases within this study were taken from a unique diabetes register which maximises the likelihood of finding an association within the study. The same is true for the Austrian study by Thurner et al [27]. In comparison, the use of the nationally representative nutrition survey by Li et al. may be under representative of the total T2DM cases within famine affected areas [23]. Context specific factors also influence findings of a famine's impact on T2DM. For example, it should be noted that the sample population examined from the Leningrad siege was largely malnourished before the siege, and remained malnourished after the siege [24] – it is hypothesised that this meant children did not experience accelerated weight gain, thought to be a mediating factor in the link between maternal undernutrition and offspring metabolic risk factors, particularly impacting on glucose-insulin metabolism[39,40]. Supporting this theory are results from offspring of the Biafran famine in Nigeria, which indicated fetal exposure to famine was associated with higher adult BMI in offspring. While a significant increase in T2DM was not apparent once adjusted for these differences in BMI, before adjustment the risk of T2DM was three times higher in exposed infants [25]. Exposed infants also showed a significant increase in impaired glucose tolerance (aOR = 1.65, 95% CI = 1.02-2.69) [25].

Our review identified three papers which consistently associated maternal overweight with T2DM, all indicated a significant impact with higher maternal weight linked to increased risk of T2DM in offspring. The largest of these, involving the HBC identified a 20% increased risk of T2DM for offspring of overweight mothers (BMI>28 kg/m^2^), along with a significant trend for increasing risk with increasing maternal weight [18].

This review found some evidence to suggest an increased risk of T2DM in offspring exposed to maternal over nutrition and under-nutrition, the impact of which appears to be dependent on unhealthy lifestyle factors in offspring. Though not included within this review, studies investigating the impact of gestational diabetes on offspring’s T2DM risk support the theory of trimester specific impacts as they show a trimester specific impact of a hyperglycemic intrauterine environment in mothers with gestational diabetes or type 1 diabetes, on the offspring’s risk of developing T2DM [41,42]. The association is also supported by findings in some populations that a decline in T2DM prevalence correlates to reductions in low-birth weight pregnancies [43,44].

This relationship between maternal nutrition status and T2DM is an important topic for future research as it could explain some of the rapidly growing prevalence rates of T2DM in LLMICs where nutrition transitions have occurred.

### Cancer

Though limited, available studies investigating the impact of maternal exposure to famine before or during pregnancy suggest a 4-fold increased risk of breast cancer and 2-fold increase in cancer related mortality for female offspring – particularly for those exposed in early gestation. For men, no significant increase in cancer related mortality was identified, in line with findings of reduced occurrence of malignant neoplasms, and specifically, a reduction in testicular cancer. There was some evidence for an increased risk of breast cancer in relation to mothers GWG. This was from a small sample however and the level of GWG linked to increased risk (11-15 kg) lies within the WHO recommendations for underweight and healthy weight mothers [13], higher levels of GWG were also not associated with offspring’s breast cancer risk. The larger HBC also found no association between maternal weight and offspring’s risk of cancer or cancer related mortality.

Amongst 25-45 year-old Swedish men it has also been found that for each 1000 g increase in birthweight the risk of cancer incidence rose 17% [45]. Findings for a reduced prevalence of testicular cancer are also consistent with an analysis of TC incidence in Scandinavian countries which shows an interruption in the increasing prevalence corresponding to men’s birth during WWII [46]. This relationship also has a plausible biological explanation as higher maternal weight is associated with increased bioavailability of sex hormones including estrogen, which has been proposed as a risk factor for testicular cancer [30].

### Chronic obstructive pulmonary diseases

Currently there are too few studies to form conclusions on the association between maternal nutrition and COPD. Although our search uncovered 32 papers investigating the impact of maternal nutrition factors on asthma, most measured this outcome in children aged 3-8 years and were excluded due to age restrictions. While the included paper suggests low maternal vitamin D status may reduce the risk of allergic asthma at 25 years of age, it should be noted that the study involved a relatively small sample with just 126 cases [32]. Results were however important as Denmark has been shown to have a high prevalence of vitamin D deficiency as well as showing a high national prevalence of COPD [47,48]. Within the same cohort maternal obesity has also been indicated as a potential risk factor for asthma in childhood measured at seven years of age [49]. Contradictory results have been noted on the role of maternal vitamin D on asthma in offspring in previous studies [32] with a 2016 systematic review failing to identify a significant association [50].

Though no papers met the inclusion criteria of the current review, some more recent studies have indicated maternal exposure is associated with an increased risk of COPD in adult offspring [51,52].

### Quality assessment

Results of assessment with the NOS (S3) found included cohort studies showed a low risk of bias for the representativeness of samples and comparators, demonstration that outcomes of interest were not present at the commencement of the study, outcome measurements and follow-up duration. The risk of bias was higher in the comparability of cohorts due to the risk of residual confounders that were not controlled for, including offspring behavioral risk factors for NCDs (diet, physical inactivity, alcohol and tobacco use), and family history of disease. The adequacy of follow-up in cohorts was also an area of potential bias with some studies reporting on only half the total cohort. Some cohorts were also limited in power due to small sample sizes within analyses. Maternal weight measurement methods and timings were mixed: some studies measured weight at only one-time point (in early or late pregnancy), and others relied on mothers’ recall years after the fact.

Publication bias may be swaying results within this review. The majority of papers come from large, high quality cohorts reducing the likelihood of publication bias at a review level. At an outcome level, two papers investigating the association between maternal BMI and CVD in men reported significant findings only in small subsets of subjects, with limited power and with contrasting findings in relation to maternal height [16,17]. However, the association was found in a much larger cohort with low risk of bias [18].

The nature of research into maternal health means the vast majority of studies take advantage of opportunistic maternal nutrition measures, such as location and timing of birth (in reference to famines), individual maternal weight measures or routine vitamin D status checks. The review failed to identify evidence to suggest any specific foods, nutrients or maternal dietary patterns impacted on an offspring’s risk of NCDs which limits the review’s capacity to inform detailed nutrition advice for mothers. Animal studies have suggested more specific dietary patterns may impact on offspring’s NCD risk, specifically protein deficiency, free fatty acid intake and diets high in “junk food” [41,53,54].

Availability of studies means the conclusions of this review apply primarily to European populations where cohort data are available. Currently there is a lack of evidence for examining the relationship between maternal nutrition and offspring NCD outcomes from low and lower-middle income countries where much of the global NCD burden lies. In relation to famine exposure this has important implications as the majority of included studies examine the impact of relatively short periods of famine, and opposed to extended states of food insecurity which can last generations in low income countries. A major limitation of observational studies included in our systematic review is also the risk of residual confounders influencing the results. While the majority of studies controlled for major confounders including socio-economic status, the offspring's BMI and smoking status, they could not control for family history of disease or offspring dietary and physical activity patterns. While ethical considerations limit the ability to conduct strict randomised controlled trials, interesting results have been obtained from sibling comparison studies where one sibling is born while mothers are classified as obese, and the other born after mothers have undergone gastric bypass to reduce their body weight and dietary intake. Though small (n = 111), this comparison found significant improvements in the siblings born after gastric bypass in relation to lower levels of severe obesity, greater insulin sensitivity and improved lipid profiles compared to those born before the gastric bypass [55]. This is an important finding as it indicates potential for prevention.

### Implications for the future

While ethical considerations restrain the potential for randomised controlled trials, findings of the current review warrant further examination in future studies and reviews. These studies should focus on the measurement of more specific dietary patterns, as well as the potential of nutrition interventions to prevent undernutrition, and excessive weight gain during pregnancy and the resulting impact on birth outcomes and long term NCD risk in offspring. There is an urgent need for such research from low and lower-middle income countries. Such results could work to inform food ration programs during humanitarian crises, as well as the provision of dietary advice within antenatal care. Investigations into these factors should align themselves to GAP NCD indicators and aim for uniformity across countries to expand the useable evidence base and strengthen the evidence case for a lifecycle approach to NCD prevention. There is also great potential to use existing databases to obtain maternal weight data with the intent to link this to offspring's NCD outcomes to add importance to this field of data.

Findings from available studies support existing WHO recommendations for nutrition interventions during antenatal care [13], namely counseling on healthy eating and healthy weight/weight gain as well as education and supplementation to increase protein and energy intake in undernourished mothers. In terms of health education, however, they suggest the impact of achieving these goals extends well beyond immediate birth outcomes and could help reduce an offspring’s susceptibility to NCDs, specifically CVD, T2DM, breast cancer and cancer related mortality in the future. Findings of the impact of famine on T2DM and CVD in offspring hold important implications for many low and lower-middle income countries undergoing rapid nutritional transitions where the next generation may have been exposed to spectrums of contrast between fetal undernutrition in gestation followed by postnatal calorie excess. On the other end of the maternal nutrition spectrum, maternal obesity consistently showed an association with an increased prevalence of NCDs. Although it is difficult to control for the impacts of shared hereditary factors, environments and offspring’s lifestyle factors – the consistency of these findings do suggest a critical window for impact during pregnancy. This is supported by findings of maternal BMIs impact on birthweight, and the subsequent association between birthweight and NCDs later in life. These findings hold important implications for both policy makers and practitioners, as it shows added importance, and urgency of promoting healthy weight, and weight gain during pregnancy.

## CONCLUSIONS

The shortage of studies identified by this review is in itself a surprising finding considering that maternal weight, and vitamin deficiencies are commonly recorded in the maternal health protocols for many countries.

While the level of evidence is not high enough to prove causation, included studies did provide relatively consistent findings to indicate both exposure to famine, and high maternal body weight may increase an offspring’s risk of CVD (particularly cerebrovascular disease) and T2DM. This relationship appears to be stronger for female offspring exposed during early gestation. In the case of famine exposure and T2DM, these diseases appear reliant upon an offspring’s exposure to contrasting nutritional environments after birth. For female offspring, both exposure to famine and maternal overweight during gestation was also associated with increased risk of cancer, specifically breast cancer. For men, exposure to famine during gestation appears to be associated with reduced risk of cancer. Though there are some indications of an association, currently there are too few studies to make conclusions on the impact of maternal nutrition on adult offspring’s COPD outcomes.

## Additional material

Online Supplementary Document
