# Supplementary material for: The impact of maternal nutrition on offspring’s risk of non-communicable diseases in adulthood: a systematic review
Source: J Glob Health. 2019 Sep 5;9(2):020405. doi: 10.7189/jogh.09.020405 (PMC6790233; doi:10.7189/jogh.09.020405)
Supplement: Online Supplementary Document [file jogh-09-020405-s001.pdf]

J Glob Health 2019; 9: 020405

Appendix S1. PRISMA Checklist

Section/topic	#	Checklist item	Reported on page #
**TITLE**			
Title	1	Identify the report as a systematic review, meta-analysis, or both.	1
**ABSTRACT**			
Structured summary	2	Provide a structured summary including, as applicable: background; objectives; data sources; study eligibility criteria, participants, and interventions; study appraisal and synthesis methods; results; limitations; conclusions and implications of key findings; systematic review registration number.	3
**INTRODUCTION**			
Rationale	3	Describe the rationale for the review in the context of what is already known.	5
Objectives	4	Provide an explicit statement of questions being addressed with reference to participants, interventions, comparisons, outcomes, and study design (PICOS).	6
**METHODS**			
Protocol and registration	5	Indicate if a review protocol exists, if and where it can be accessed (e.g., Web address), and, if available, provide registration	8

		information including registration number.	
Eligibility criteria	6	Specify study characteristics (e.g., PICOS, length of follow-up) and report characteristics (e.g., years considered, language, publication status) used as criteria for eligibility, giving rationale.	8-9
Information sources	7	Describe all information sources (e.g., databases with dates of coverage, contact with study authors to identify additional studies) in the search and date last searched.	8
Search	8	Present full electronic search strategy for at least one database, including any limits used, such that it could be repeated.	S2
Study selection	9	State the process for selecting studies (i.e., screening, eligibility, included in systematic review, and, if applicable, included in the meta-analysis).	9
Data collection process	10	Describe method of data extraction from reports (e.g., piloted forms, independently, in duplicate) and any processes for obtaining and confirming data from investigators.	9
Data items	11	List and define all variables for which data were sought (e.g., PICOS, funding sources) and any assumptions and simplifications made.	9
Risk of bias in individual studies	12	Describe methods used for assessing risk of bias of individual studies (including specification of whether this was done at the study or outcome level), and how this information is to be used in any data synthesis.	10
Summary measures	13	State the principal summary measures (e.g., risk ratio, difference in means).	10
Synthesis of results	14	Describe the methods of handling data and combining results of studies, if done, including measures of consistency (e.g., I^2) for each meta-analysis.	10

Section/topic	#	Checklist item	Reported on page #
Risk of bias across studies	15	Specify any assessment of risk of bias that may affect the cumulative evidence (e.g., publication bias, selective reporting within studies).	36-37
Additional analyses	16	Describe methods of additional analyses (e.g., sensitivity or subgroup analyses, meta-regression), if done, indicating which were pre-specified.	-
**RESULTS**			
Study selection	17	Give numbers of studies screened, assessed for eligibility, and included in the review, with reasons for exclusions at each stage, ideally with a flow diagram.	11
Study characteristics	18	For each study, present characteristics for which data were extracted (e.g., study size, PICOS, follow-up period) and provide the citations.	13-19, 22-24, 26-28, 30
Risk of bias within studies	19	Present data on risk of bias of each study and, if available, any outcome level assessment (see item 12).	S3
Results of individual studies	20	For all outcomes considered (benefits or harms), present, for each study: (a) simple summary data for each intervention group (b) effect estimates and confidence intervals, ideally with a forest plot.	13-19, 22-24, 26-28, 30
Synthesis of results	21	Present results of each meta-analysis done, including confidence intervals and measures of consistency.	-
Risk of bias across studies	22	Present results of any assessment of risk of bias across studies (see Item 15).	36-37
Additional analysis	23	Give results of additional analyses, if done (e.g., sensitivity or subgroup analyses, meta-regression [see Item 16]).	-
**DISCUSSION**			

Summary of evidence	24	Summarize the main findings including the strength of evidence for each main outcome; consider their relevance to key groups (e.g., healthcare providers, users, and policy makers).	31-39
Limitations	25	Discuss limitations at study and outcome level (e.g., risk of bias), and at review-level (e.g., incomplete retrieval of identified research, reporting bias).	36-37
Conclusions	26	Provide a general interpretation of the results in the context of other evidence, and implications for future research.	38-39
**FUNDING**			
Funding	27	Describe sources of funding for the systematic review and other support (e.g., supply of data); role of funders for the systematic review.	2

Appendix S2. Search Terms

# ▲	Searches
1	Pregnancy/
2	exp Pregnancy Trimesters/
3	Pregnant Women/
4	preconception care/ or prenatal care/
5	Maternal Health/
6	(pregnant or pregnancy or gestational or preconcept* or pre-concept* or prepregnan* or pre-pregnan*).ti.
7	Postpartum Period/
8	Lactation/
9	Breast Feeding/
10	Bottle Feeding/
11	Infant Food/
12	Weaning/
13	(postnatal or post-natal or postpartum or post-partum).ti,ab.
14	(lactation or lactating or breastfeed* or breast feed* or breast fed or breastfed or bottle feed* or bottle fed or bottlefeed or bottlefed or

infant feeding or weaning or weaned).ti.

15 1 or 2 or 3 or 4 or 5 or 6 or 7 or 8 or 9 or 10 or 11 or 12 or 13 or 14 or 15

16 Weight Gain/

17 overweight/ or obesity/ or obesity, morbid/

18 Overnutrition/

19 (obes* or overweight or adipos*).ti.

20 Weight Loss/

21 Thinness/

22 malnutrition/ or starvation/

23 exp Deficiency Diseases/

24 exp *Diet/

25 exp *Food/

26 Sodium Chloride, Dietary/

27 exp Drinking Behavior/

28 exp *Beverages/

29 (diet or nutrition* or undernutrition or malnutrition or starvation or famine or underweight or thin or slim).ti.

30 ((energy or calor* or food or drink*) adj2 (intake or consumption)).ti,ab.

31 ((healthy or healthful) adj eating).ti,ab.

32	(fruit? or vegetable? or salt or sodium or vitamin? or mineral? or nutrient? or micronutrient?).ti.
33	((fruit? or vegetable? or salt or sodium or vitamin? or mineral? or nutrient? or micronutrient?) adj3 (intake or consum* or diet*)).ti,ab.
34	((vitamin? or mineral? or diet* or nutrition* or nutrient? or micronutrient?) adj3 (deficiency or deficient)).ti,ab.
35	(artificial sweetener? or artificial sweetening or sugar substitute? or sweetening agent? or corn syrup? or fructose).ti,ab.
36	(alcohol* or drink*).ti.
37	(alcohol* adj3 (drink* or factor* or pattern* or habit* or consum* or unhealthy)).ti,ab.
38	(((carbonated or sugar* or sweetend or soft) adj2 (drink? or bevarage?)) or soda? or fruit juice?).ti,ab.
39	Body Mass Index/
40	(body mass or bmi).ti.
41	16 or 17 or 18 or 19 or 20 or 21 or 22 or 23 or 24 or 25 or 26 or 27 or 28 or 29 or 30 or 31 or 32 or 33 or 34 or 35 or 36 or 37 or 38 or 39 or 40
42	15 and 41
42	((maternal or mother*) adj2 (weight or bodyweight or overweight or obes* or adipos* or bmi or body mass or underweight or undernutrition or malutrition or starvation or thin* or slim or excess weight or excess fat)).ti,ab.

43	((pregnant or pregnancy or prepregnan* or pre-pregnan* or gestational) adj2 (weight or bodyweight or overweight or obes* or adipos* or bmi or body mass or underweight or undernutrition or malnutrition or starvation or thin* or slim or excess weight or excess fat)).ti,ab.
44	((preconcept* or pre-concept*) adj2 (weight or bodyweight or overweight or obes* or adipos* or bmi or body mass or underweight or undernutrition or malnutrition or starvation or thin* or slim or excess weight or excess fat)).ti,ab.
45	((prenatal or pre-natal or prepartal or prepartum or pre-partal or pre-partum or antenatal or antepartum or ante-natal or ante-partum) adj2 (weight or bodyweight or overweight or obes* or adipos* or bmi or body mass or underweight or undernutrition or malnutrition or starvation or thin* or slim or excess weight or excess fat)).ti,ab.
46	((maternal or mother*) adj2 (diet* or nutrition*)).ti,ab.
47	((pregnant or pregnancy or prepregnan* or pre-pregnan* or gestational) adj2 (diet* or nutrition*)).ti,ab.
48	((preconcept* or pre-concept*) adj2 (diet* or nutrition*)).ti,ab.
49	((prenatal or pre-natal or prepartal or prepartum or pre-partal or pre-partum or antenatal or antepartum or ante-natal or ante-partum) adj2 (diet* or nutrition*)).ti,ab.
50	maternal nutritional physiological phenomena/ or prenatal nutritional physiological phenomena/

51	((postnatal or post-natal or postpartum or post-partum) adj2 (weight or bodyweight or overweight or obes* or adipos* or bmi or body mass or underweight or undernutrition or malutrition or starvation or thin* or slim or excess weight or excess fat)).ti,ab.
52	((lactation or lactating or breastfeed* or breast feed* or breast fed or breastfed or bottle feed* or bottle fed or bottlefeed or bottlefed or infant feeding or weaning or weaned) adj2 (weight or bodyweight or overweight or obes* or adipos* or bmi or body mass or underweight or undernutrition or malnutrition or starvation or thin* or slim or excess weight or excess fat)).ti,ab.
53	((postnatal or post-natal or postpartum or post-partum) adj2 (diet* or nutrition*)).ti,ab.
54	((lactation or lactating or breastfeed* or breast feed*) adj2 (diet* or nutrition*)).ti,ab.
55	Infant Nutritional Physiological Phenomena/
56	42 or 43 or 44 or 45 or 46 or 47 or 48 or 49 or 50 or 51 or 52 or 53 or 54 or 55
57	exp child/ or infant/
58	(infant? or infancy or child* or preschool* or pre-school* or toddler* or pediatric* or paediatric or juvenile or offspring).ti.
59	57 or 58
60	overweight/ep or obesity, morbid/ep

61 exp Body Composition/

62 growth/ or body size/ or body height/ or body weight/ or birth weight/

63 Blood Pressure/

64 Hypertension/

65 Cholesterol/bl [Blood]

66 Triglycerides/bl [Blood]

67 Lipids/bl [Blood]

68 exp Hyperlipidemias/

69 Blood Glucose/

70 Insulin/bl [Blood]

71 Puberty, Precocious/

72 Mortality/

73 Asthma/

74 exp Pulmonary Disease, Chronic Obstructive/

75 exp *Neoplasms/

76 (obes* or overweight* or bmi or body mass).ti.

77 (growth or height or body composition or body fat or body weight or bodyweight or weight gain or muscle mass).ti.

78 (blood pressure or hypertens*).ti.

79 ((blood or plasma or serum) adj2 (lipid* or cholesterol or

	triglyceride*)).ti,ab.
80	(hypercholesterol?emi* or hyperlipid?emi?).ti.
81	(lipid* or cholesterol or triglyceride*).ti.
82	((blood or plasma or serum) adj2 glucose).ti,ab.
83	(insulin adj2 (sensitivity or tolerance or growth factor)).ti,ab.
84	(asthma* or ((lung or respiratory or pulmonary) adj function)).ti,ab.
85	(chronic adj2 (lung oe pulmonary or bronchitis)).ti,ab.
86	(cancer? or neoplas* or carcinoma? or leukaemia? or leukemia? or lymphoma? or sarcoma? or malignanc*).ti.
87	(mortality or death?).ti.
88	60 or 61 or 62 or 63 or 64 or 65 or 66 or 67 or 68 or 69 or 70 or 71 or 72 or 73 or 74 or 75 or 76 or 77 or 78 or 79 or 80 or 81 or 82 or 83 or 84 or 85 or 86 or 87
89	56 and 59 and 88
90	Pediatric Obesity/
91	Child Mortality/ or Infant Mortality/
92	Prenatal Exposure Delayed Effects/
93	((infant? or infancy or child* or preschool* or pre-school* or toddler* or pediatric* or paediatric or juvenile or offspring) adj2 (obes* or overweight)).ti,ab.

94	((infant? or infancy or child* or preschool* or pre-school* or toddler* or pediatric* or paediatric or juvenile or offspring) adj5 (lipid* or cholesterol or triglyceride* or hypercholesterol?emi* or hyperlipid?emi?)).ti,ab.
95	((infant? or infancy or child* or preschool* or pre-school* or toddler* or pediatric* or paediatric or juvenile or offspring) adj5 (blood glucose or insulin)).ti,ab.
96	((infant? or infancy or child* or preschool* or pre-school* or toddler* or pediatric* or paediatric or juvenile or offspring) adj5 (blood pressure or hypertens*)).ti,ab.
97	((infant? or infancy or child* or preschool* or pre-school* or toddler* or pediatric* or paediatric or juvenile or offspring) adj5 (growth or height or body composition or body fat or body weight or bodyweight or weight gain or muscle mass)).ti,ab.
98	((postnatal or post-natal or postpart* or post-part* or "catch up") adj2 (growth or weight gain)).ti,ab.
99	((early or precocious or premature) adj2 puberty).ti,ab.
100	((infant? or infancy or child* or preschool* or pre-school* or toddler* or pediatric* or paediatric or juvenile or offspring) adj2 (mortality or death?)).ti,ab.
101	((infant? or infancy or child* or preschool* or pre-school* or toddler* or pediatric* or paediatric or juvenile or offspring) adj5 (cancer? or neoplas* or carcinoma? or leukaemia? or leukemia? or lymphoma? or sarcoma? or malignanc*)).ti,ab.

102 90 or 91 or 92 or 93 or 94 or 95 or 96 or 97 or 98 or 99 or 100 or 101

103 42 and 102

105 exp animals/ not humans.sh.

106 (rat or rats or mouse or mice or murine or rodent?).ti.

107 105 and 106

108 103 not 107

Appendix S3. Quality Assessment using Newcastle Ottawa Scale

Cardiovascular Disease

Reference (year). Country.	Representativeness of the exposed cohort	Selection of the non-exposed cohort	Ascertainment of exposure	Demonstration that outcome of interest was not present at start of study	Comparability of cohorts on the basis of the design or analysis	Assessment of outcome	Was follow-up long enough for outcomes to occur	Adequacy of follow-up of cohorts	Additional Bias
Bhattacharya S, et al. [15](2015) Scotland	A*	A*	A*	A*	B*	B*	A*	C	Large portion of original cohort not included as only one measure of maternal weight taken.
	All registered births	Categories of Gestational Weight Gain defined	Secure record	Records from birth	Adjusts for clustering, not for environmental differences	record linkage	46 years	47% loss, no explanation for 3852	
Bygren LO, et al.[20] (2000).	A*	B*	B	A*	B*	B*	A*	B	
	Taken from all	Taken from	Based on	Records from	Adjustment for	Mixed	70 years	Only 53% of	Assumed

Sweden	births	historical records	historical references of crop supply	birth	age and civil status	recording/ terminology on death certificates		mortality cases had a specified cause	exposure, cannot fully control for other risk factors.
Eriksson JG, et al. [16] (2011). Finland	A*	A*	A*	A*	B*	A*	A*	A*	Low numbers within each measure of birthweight according to mothers BMI.
	Helsinki Birth Cohort- all births from 1932-44	Taken from same cohort	Maternal hospital records	Records from birth	Adjustment for social class not adult lifestyle, body weight	Hospital admissions and records	Median age0.8 years	Representative of whole cohort	
Reynolds RM, et al.[14] (2013). United Kingdom	A*	A*	A*	B*	B*	A*	A*	B*	Cannot fully control for shared genetic and postnatal lifestyle influences in childhood or adulthood
	All birth records	Matched from same population	ANC clinic visits in database	Followed from birth	Adjusted for maternal age at delivery, gestation when weight was measured, social	Hospital records	34-61 years	Convenient sample from database. Only 49% of exposed cohort, 40% non exposed cohort	

					class, parity, sex of offspring, gestation at delivery, birth weight, and current age of offspring.			assessed	behavioural risks.
Painter RC, et al.[19] (2006)<br><br>Netherlands	A*<br>Dutch Famine birth Cohort	B<br>Matched from same population	B*<br>Based on location and timing of birth	A*<br>Followed from birth	B*<br>Adjusted for gender, size at birth, BMI, smoking, SES	A*<br>Hospital examination	A*<br>50-58 years	C<br>only 49% of cohorts assessed	Selective participation of persons who were fit enough to attend the clinic, may underestimate the effect.
Eriksson JG, et al.[18] (2014).	A*<br>Helsinki Birth	A*<br>National	A*<br>Maternal height	A*<br>Followed from	A*<br>Adjusted for	A*<br>Hospital	A*<br>70+ years	A*<br>Full sample	Not adjusted for offspring adult

Finland	Cohort- all births from 1932-44	statistics	and weight recorded before delivery	birth	gender, year of birth, socioeconomic status in childhood and adult life, educational attainment, and income.	records		evaluated	lifestyle.
Van Abeelen AF, et al.[21](2012). Netherlands	A*	A*	B*	A*	B*	A*	A*	A*	Not adjusted for offspring behavioural risk factors.
	Dutch Famine Birth Cohort	Unexposed subjects from same community	Location of birth	Followed from birth	Adjusted for gender, date of birth, birth weight	Hospital records	Same methods	88%	
Ekamper P, et al.[22] (2015).	A*	A*	B*	A*	A*	A*	A*	A*	No information on smoking patterns Women not
	All surviving males of Dutch Winter	Random sample of same population born	Based on location of birth	Followed from birth	Adjusted for time and place	National mortality	63 years	All available	

Netherlands	Famine Cohort	outside of famine area			of birth	records			included.
Forsen T, et al.[17] (1997). Finland	A*<br>Men born in Helsinki University Central Hospital between 1924-1933	A*<br>National statistics	A*<br>Hospital records of maternal weight at labour	A*<br>Records from birth	B*<br>Not controlled for SES, adult BMI	A*<br>National records	A*<br>Same methods	A*<br>92% of cohort traced	Geographically restricted and conducted in an area with lower CHD than national average.

Type 2 Diabetes Mellitus

Reference (year). Country.	Representativeness of the sample	Sample Size	Non-respondents	Ascertainment of Exposure	Comparability of outcome groups	Assessment of outcome	Statistical test	Additional Bias
Ly, Y et al	A*	A*	A*	B*	B*	B*	A*	Assumption that the residents

[23](2010).<br><br>China<br><br>Cross Sectional	Taken from national survey	Justified and satisfactory	>90% in the majority of villages	Recorded birth location	Adjusted for PA, alcohol, smoking, family history, age and sex but not diet	Validated diagnosis with T2DM	Yes	were born in the same area they lived. Those exposed to famine during gestation were likely exposed during childhood also.
Stanner et al [24](1997)	B*	B	A*	B	B*	A*	A*	Only 44% of subjects analysed
Russia<br><br>Cross Sectional	Record linkage based on Society of Children of the Siege (birth register)	Only 44% of identified subjects included	Born in same time period outside of Leningrad	half of controls invited from kerotomy clinic, though no significant differences found these results were not reported	Control differences may be apparent but controlled for sex, obesity, smoking and CHD	Secure record- birth registration	Yes	
Thurner S, et	A*	A*	A*	B*	B*	A*	A*	

al.[29] (2013). Austria Cross Sectional	Whole nation sample	Justified and satisfactory	All Austrian patients included	Recorded birth location	Adjusted for date of birth, residency, and sex of the subjects. Not adult behavioural risk factors	Diagnosed and treated diabetes	Yes	
Lumey, LH et al[28] (2015). Ukraine Cross sectional	A*	A*	A*	B*	B*	A*	A*	Potential biased by differences in survival or loss to follow-up in specific exposure groups.
	All diagnosed diabetics on Ukraine register	Justified and satisfactory	Selected from matched areas not exposed to famine	Non validated method but methods comprehensively described	Adjusted for gender, but not for adult factors	Diagnosed and treated for T2DM	Yes	
Dabelea D, et al. [31](2008). USA	B*	B	A*	A*	A*	D	A*	Recall bias in maternal obesity possible. Only 54% case and 49% control sample involvement
	Sample of early T2DM cases taken from two networks	Convenient sample	Selected from primary care offices in same	Confirmed non-diabetics	Adjusted for perinatal and SES factors	self report of weight	Yes	

Case Control	of health care providers		region					

Reference (year). Country.	Representativeness of the exposed cohort	Selection of the non-exposed cohort	Ascertainment of exposure	Demonstration that outcome of interest was not present at start of study	Comparability of cohorts on the basis of the design or analysis	Assessment of outcome	Was follow-up long enough for outcomes to occur	Adequacy of follow-up of cohorts	Additional Bias
Fall CH, et al.[30] (1998). India	B*	A*	B*	A*	B*	A*	A*	C*	Limited power, small sample
	Small sample from one hospital	From same sample	Maternal weight measured at different stages of pregnancy	Followed from birth	Adjusted for age, sex and BMI. Not family history or adult lifestyle factors	Diagnosed T2DM	39-60 years	Only 40% had maternal weight measurements available	
Hult M, et al. [25](2010).	B*	A*	B*	B	B*	A*	A*	B*	Limited to Igbo ethnicity. Lack of birth weight data
	Selection of	From same	Place and year	Sample	Adjusted for BMI	Diagnosed	37-43	90% of	

Nigeria	participants from shops in six major market places	population, born in different years	of birth used as a proxy for exposure	selection may be bias	not gender or adult lifestyle	T2DM		approached subjects	and the inability to separate effects of fetal and infant famine.

Cancer

Reference (year). Country.	Representativeness of the exposed cohort	Selection of the non-exposed cohort	Ascertainment of exposure	Demonstration that outcome of interest was not present at start of study	Comparability of cohorts on the basis of the design or analysis	Assessment of outcome	Was follow-up long enough for outcomes to occur	Adequacy of follow-up of cohorts	Additional Bias
Aschim EL, et al. (2005). Norway	A*	A*	A*	A*	A*	B	A*		maternal weight only measured a few days
	Clinical diagnosis with testicular cancer	Large sample from national hospital	All men without testicular	Confirmed testicular cancer free	All men from national sample	Weight measured a few days	Matched measurement		

Cross-sectional			cancer			before birth			before
Reference (year). Country.	Is the case definition adequate	Representativeness of cases	Selection of controls	Definition of controls	Comparability of cases and controls	Ascertainment of exposure	Ascertainment of exposure-controls	Non-response rate	
Painter RC, et al.[19] (2006). Netherlands	A*	A*	B*	A*	A*	A*	A*	B	Low power and relatively young cohort.
	Women from Dutch Famine Cohort	Same population born outside of famine periods	Place and year of birth used as a proxy	Followed from birth	Adjusted for adult, birth and maternal risk factors	Diagnosed breast cancer	57-59 years	Only 62% of eligible women in the cohort participated	
Van Abeelen AF, et al. [21](2012). Netherlands	A*	A*	B*	A*	B*	A*	A*	A*	Not adjusted for offspring behavioural risk factors
	Dutch Famine Birth Cohort	Unexposed subjects from same community	Location of birth	Followed from birth	Adjusted for gender, date of birth, birth weight	Hospital records	Same methods	88%	
Sanderson M, et al.[33] (1998).	A*	A*	A*	A*	A*	B*	A*	C	Interview of mothers
	Diagnosed breast	Representative	Age matched	No history of	Adjusted for	Structured	Same as Cases	Only 53.9%	

USA<br><br>Case Control	cancer	series of cases	community controls	disease	age, birth year, family history, age of menarche, menopauseal status, pregnancies, oral contraceptive use, BMI, maternal age, smoking, birthweight,	interview		of living cases, 28% deceased cases and 50.3% of control mothers responded	conducted years after pregnancy. Potential selection bias, and low of statistical power.

COPD

Reference (year).	Representativeness of the exposed	Selection of the non-	Ascertainment of exposure	Demonstration that outcome	Comparability of cohorts on	Assessment of outcome	Was follow-up	Adequacy of follow-up of	Additional Bias

Country.	cohort	exposed cohort		of interest was not present at start of study	the basis of the design or analysis		long enough for outcomes to occur	cohorts	
Hansen S, et al.[34] (2015)<br><br>Denmark	A*	B	A*	A*	B*	B*	A*	C	relied on a single 25(OH) D measurement, which might
	Danish Fetal Origins Cohort 1988-1989	Mothers with no vitamin D level available	Blood serum samples in 3rd Trimester	Followed from birth	Adjusted for self reported maternal education, pre-pregnancy BMI, smoking and offspring height	Clinical examination (n-410) and self report (n-641)	20 years	Only 70% of cohort	
